# Belongingness and School Refusal in Adolescents: Psychometric Validation and Identification of Risk Profiles

**DOI:** 10.3390/bs16071225

**Published:** 2026-07-18

**Authors:** María Pérez-Marco, Carolina Gonzálvez, Andrea Fuster, Ángel Amat, Alba Lorenzo-Rumbo, Graciela Arráez-Vera

**Affiliations:** Department of Developmental Psychology and Didactics, Faculty of Education, University of Alicante, Carr. de San Vicente del Raspeig, s/n, 03690 San Vicente del Raspeig, Spain; mperez.marco@ua.es (M.P.-M.); andrea.fuster@ua.es (A.F.); angel.amat@ua.es (Á.A.); alba.lorenzo@ua.es (A.L.-R.); graciela.arraez@ua.es (G.A.-V.)

**Keywords:** general belongingness, school refusal, adolescents, latent profile analysis, psychometric properties

## Abstract

Belongingness is a fundamental psychological need associated with adolescents’ emotional well-being, school engagement, and attendance. Despite the relevance of belongingness for adolescent adjustment, no validated measure of general belongingness is currently available for Spanish adolescents, and little is known about how different belongingness patterns relate to school refusal behaviour. This study provides the first validation of the General Belongingness Scale (GBS) in a sample of Spanish adolescents (*N* = 690; 12–17 years) and examines latent belongingness profiles and their links to school refusal. Confirmatory Factor Analysis supported a correlated two-factor structure (Inclusion and Exclusion) with satisfactory reliability, and measurement invariance across gender was established at the configural, metric, and scalar levels. Convergent validity was supported by meaningful associations between GBS dimensions and school refusal factors. Latent Profile Analysis identified four profiles: Highly Excluded, Socially Disconnected, Mixed Belonging, and Highly Included, which differed significantly in school refusal, with the *Highly Excluded* profile showing the highest risk. These findings support the psychometric adequacy of the GBS and identify belongingness as a meaningful socio-emotional factor associated with school refusal risk, with potential implications for early identification and preventive interventions aimed at promoting school attendance and well-being.

## 1. Introduction

A sense of belonging has been widely recognised as a cornerstone of psychological adjustment and human well-being. Classic and contemporary theoretical approaches consistently identify belongingness as a fundamental psychological need essential for healthy functioning across the lifespan (Baumeister & Leary, 1995; Martela, 2024; Maslow, 1968). It reflects the experience of feeling valued, accepted and connected within one’s social environment, encompassing both affective comfort and perceived interpersonal fit (Hagerty et al., 1992; Yildiz, 2017). When satisfied, belongingness contributes to security, life satisfaction and social adjustment (Lavigne et al., 2011), whereas its frustration through exclusion or rejection is associated with loneliness, emotional distress, internalising symptoms and reduced self-esteem (Baumeister et al., 2007; Hagerty & Williams, 2010; Yildirim et al., 2023). During adolescence, marked by heightened sensitivity to peer relations and social evaluation, belongingness plays a particularly protective role, buffering against loneliness, depressive symptoms and social disconnection (Allen & Bowles, 2012; Baskin et al., 2010; Fernández-Sogorb et al., 2026; Gallé-Tessonneau et al., 2019; Yildirim et al., 2023; Yildiz, 2016).

Given its relevance, various instruments have been developed to assess belongingness or related constructs. However, most existing measures are context-specific, focusing exclusively on school, family or peers (Malone et al., 2012). Examples include the Sense of Belonging Instrument (Hagerty & Patusky, 1995), the Social Connectedness and Social Assurance Scales (Lee & Robbins, 1995), the Need to Belong Scale (Leary et al., 2013), the Adolescents’ Societal Belongingness Scale (Ozdemir & Bayram, 2023), the School Belonging Scales (Arslan & Duru, 2017; Yildirim et al., 2023) and the Sense of Belonging to School Scale (Akar-Vural et al., 2013). Despite their utility, these instruments provide only partial assessments of the construct by examining specific social spheres rather than an overarching sense of belonging; they also rely heavily on negatively worded items (Checa & Oberst, 2022; Malone et al., 2012) and often lack validation in adolescent populations. Consequently, there remains a need for a psychometrically robust measure capable of evaluating both inclusion and exclusion processes across multiple domains of social life and validated specifically in adolescent populations.

The General Belongingness Scale (GBS; Malone et al., 2012) was developed to address these limitations. This 12-item instrument assesses achieved belongingness through six inclusion and six exclusion items, providing a balanced and comprehensive measure. Initial validation studies supported a stable two-factor structure, Acceptance/Inclusion and Rejection/Exclusion factors, with excellent reliability (α = 0.92) and high inter-factor correlation, reflecting the unified yet multifaceted nature of belongingness. Since its development, the GBS has been adapted in several cultural contexts. In Turkey, validations with young adults (Satici & Gocet-Tekin, 2016) and adolescents (Yildiz, 2017) confirmed the two-factor model and demonstrated satisfactory reliability. Belongingness measured through the GBS was significantly associated with loneliness, life satisfaction and attachment to parents and peers (Yildiz, 2017), reinforcing its relevance during adolescence.

More recently, Checa and Oberst (2022) conducted the first Spanish validation of the GBS using an adult sample. Their study evaluated four competing confirmatory models, finding that the best-fitting solution was a one-factor structure with an additional method factor accounting for reversed-worded items. This model showed excellent reliability (α = 0.92; CR = 0.91) and demonstrated strong measurement invariance across gender, with no significant latent mean differences between men and women. However, the authors emphasised the need for further research examining construct validity in other age groups such as Spanish adolescents, a population in which belongingness plays a critical developmental role (Fernández-Sogorb et al., 2026; Gallé-Tessonneau et al., 2019).

However, apart from Yildiz (2017), no validation has been reported in adolescent samples of other countries. This gap underscores the novelty of validating the GBS in Spanish adolescents. Considering that belongingness in adolescence is linked to key indicators of psychological health, such as life satisfaction, attachment, emotional well-being and internalising distress (Yildirim et al., 2023; Yildiz, 2016, 2017), a validated measure in this population is essential. Furthermore, in the educational context, diminished belongingness has been associated with negative school-related outcomes, including anxiety, peer difficulties and school disengagement, which may relate to problematic behaviours (Hamilton, 2024). Understanding how adolescents perceive inclusion and exclusion may, therefore, offer valuable insights into patterns of emotional vulnerability and school adjustment.

One area where belongingness may play a particularly salient role is school refusal, defined as a type of school attendance problem that is characterised by a difficulty attending or remaining in school due to emotional distress (Havik & Ingul, 2022; Heyne et al., 2019). Theoretical models suggest that adolescents with low belongingness experience heightened anxiety, interpersonal discomfort and withdrawal, core risk factors for school refusal (Gubbels et al., 2019; Hamilton, 2024; Leduc et al., 2024). Although empirical research directly linking general belongingness to school refusal is absent, belongingness is strongly related to internalising symptoms (Yildirim et al., 2023), which are well-established predictors of school refusal (Gonzálvez et al., 2018, 2020a, 2020b). Notably, the only study addressing both constructs is the theoretical analysis by Hamilton (2024), who proposed that low belongingness increases vulnerability to emotional-based school avoidance via negative affect, anxiety and social disconnection. However, this work remains conceptual and theoretical and lacks empirical testing, highlighting the need for research that directly explores this relationship.

A further gap concerns the absence of person-centred analyses examining heterogeneity in adolescents’ belongingness experiences. Latent Profile Analysis (LPA) can identify subgroups of adolescents characterised by different patterns of inclusion and exclusion, offering a nuanced understanding beyond variable-centred approaches (Song & Kim, 2019). Such profiles may elucidate how belongingness configurations relate to emotional, behavioural and academic functioning. Yet, no studies have identified latent belongingness profiles using the GBS nor examined whether these profiles differ in school refusal tendencies, a surprising omission given the centrality of belongingness to adolescent socio-emotional development and the value of profile-based approaches for targeted intervention.

### The Present Study

The present study addresses these empirical and conceptual gaps by conducting the validation of the General Belongingness Scale (GBS) in Spanish adolescents, while simultaneously identifying meaningful belongingness profiles and examining their relationship with school refusal. Specifically, the study has four primary objectives: (1) to examine the psychometric properties of the GBS in Spanish adolescents; (2) to evaluate measurement invariance across gender; (3) to assess convergent validity by analysing associations between belongingness dimensions and school refusal factors; and (4) to identify latent belongingness profiles using LPA and determine whether these profiles differ meaningfully in school refusal behaviour.

Based on robust evidence from the original development (Malone et al., 2012), cross-cultural validations in adolescents (e.g., Arslan, 2021), and the Spanish adult adaptation (Checa & Oberst, 2022), it was hypothesised that the two-factor structure of Acceptance/Inclusion and Rejection/Exclusion would be replicated (Hypothesis 1) and would demonstrate adequate internal consistency (Hypothesis 2). Given previous findings showing structural stability across gender (Checa & Oberst, 2022), measurement invariance across gender was also expected (Hypothesis 3). Furthermore, grounded in research linking exclusion to internalising symptoms (Arslan, 2021; Hamilton, 2024; Yildirim et al., 2023) and internalising symptoms to school refusal (Gonzálvez et al., 2018; Heyne et al., 2019; Havik & Ingul, 2022), higher exclusion was predicted to correlate positively, and higher inclusion negatively, with school refusal (Hypothesis 4).

From a person-centred perspective, previous latent profile studies on belongingness are scarce, but the heterogeneous configurations of inclusion and exclusion are likely to emerge. Thus, it was anticipated that at least two distinct belongingness profiles would be identified: a profile with high exclusion and low inclusion scores and another profile with high inclusion and low exclusion punctuations (Hypothesis 5). Finally, drawing on theoretical models linking belongingness with emotional vulnerability and avoidance-based behaviours (Arslan, 2021; Gonzálvez et al., 2018; Hamilton, 2024; Heyne et al., 2019; Yildirim et al., 2023), adolescents in profiles characterised by high exclusion and low inclusion were expected to exhibit the highest levels of school refusal, whereas those in high inclusion and low exclusion profiles would show the lowest (Hypothesis 6).

## 2. Materials and Methods

### 2.1. Participants

Participants were recruited using a random cluster sampling strategy designed to ensure representativeness across the main geographic areas of the province of Alicante (central, northern, southern, eastern, and western zones). The sampling procedure followed a multistage structure. In the first stage, high schools served as primary sampling units, and one or two public or private institutions were randomly selected within each geographic area, resulting in a total of six schools. In the second stage, classrooms were randomly sampled within each participating school, covering different academic levels. Three classrooms per institution were selected, with representation from one or two class groups within each grade from the first to the fourth year of compulsory secondary education, as well as post-compulsory levels.

Using this approach, 802 students initially took part in the study. Of these, 112 were excluded from the final dataset for the following reasons: 83 because written parental consent was not received within the required timeframe, 18 due to insufficient comprehension of the questionnaire language, 3 because they presented identified special educational needs that did not meet the study inclusion criteria, and 1 due to an invalid response pattern. The remaining 697 cases were retained for the preliminary data screening. Subsequently, seven multivariate outliers were identified using Mahalanobis distance and removed prior to the main analyses, resulting in a final sample of 690 adolescents. The final analytic sample, therefore, consisted of 690 adolescents aged 12 to 17 years (*M_age_* = 14.21; *SD* = 1.42), of whom 50.4% self-identified as boys, 48.3% as girls, and 1.3% as another gender (see Table 1). The distribution of participants across age and gender groups was statistically homogeneous, as indicated by the Chi-square test (χ^2^ = 16.53, *p* = 0.09).

### 2.2. Instruments

General Belongingness Scale (GBS; Malone et al., 2012). The GBS is a 12-item self-report measure designed to assess individuals’ overall sense of belongingness. Items are rated on a 7-point Likert scale ranging from 0 (strongly disagree) to 6 (strongly agree). The instrument comprises six items reflecting perceived acceptance and inclusion (e.g., “When I am with other people, I feel included”; “I feel accepted by others”) and six items capturing feelings of rejection or exclusion (e.g., “I feel like an outsider”; “I feel isolated from the rest of the world”). For the present study, we employed the Spanish version developed and validated in an adult population by Checa and Oberst (2022). The GBS in adolescents sample confirmed adequate psychometric properties (Inclusion: α = 0.81, Ω = 0.81; Exclusion: α = 0.78, Ω = 0.78), including strong composite reliability and invariance across gender.

School Refusal Evaluation Scale (SCREEN; Gallé-Tessonneau & Gana, 2019) is an 18-item self-report questionnaire assessing four functional dimensions of school refusal among young people aged 11 to 16 years. Respondents indicate the extent to which each statement describes their experience on a 5-point Likert scale (1 = Not at all like me; 5 = Very much like me). The scale includes four factors: FI) Anxious anticipation (e.g., “I’m afraid to go to school”), FII) Difficult transition (e.g., “In the morning, I don’t want to go to school”), FIII) Interpersonal discomfort (e.g., “I’m scared of doing a bad job in class”), and FIV) School avoidance (e.g., “I’m frequently absent because I don’t feel well”). In this study, the Spanish SCREEN was used (Pérez-Marco et al., 2026), which has demonstrated robust psychometric properties for the Spanish adaptation, including adequate internal consistency estimates across the four factors: FI: α = 0.74, Ω = 0.74, FII: α = 0.73, Ω = 0.74, FIII: α = 0.71, Ω = 0.73, FIV: α = 0.73, Ω = 0.73.

### 2.3. Procedure

The Spanish version of the General Belongingness Scale (GBS) originally developed for adults was administered in its original form (Checa & Oberst, 2022). No modifications were made to the wording, content, number of items, or response format for use with adolescents. This decision was based on the age-neutral nature of the construct and the general wording of the items, which describe universal experiences of interpersonal belongingness rather than age-specific situations. Consequently, the present study aimed to evaluate whether the original Spanish version demonstrates adequate psychometric properties in a Spanish adolescent sample.

In addition, randomly selected schools across different areas of the province of Alicante were contacted and informed about the aims and procedures of the study. Ethical approval was granted by the University of Alicante (UA-2023-03-07), and written informed consent was obtained from the parents or legal guardians of all participating students, in accordance with the principles of the Declaration of Helsinki (World Medical Association, 2013). In addition to obtaining written informed consent from parents or legal guardians, all participating adolescents provided their own informed assent prior to completing the questionnaires. Participation was entirely voluntary, and students were informed that they could withdraw from the study at any time without any consequences. Finally, the GBS and SCREEN were administered during regular school hours, and participation was voluntary and anonymous. A member of the research team was present during administration to clarify procedural aspects and ensure that students fully understood the survey process.

### 2.4. Data Analysis

Prior to conducting the main analyses, preliminary data screening was performed. Missing responses were not present, as the survey platform (Google Forms) required all items to be completed sequentially. Even though data were primarily collected via Google Forms with forced-response settings, ensuring no missing data in the online dataset, in some school contexts where digital access was not available, a small number of paper-and-pencil questionnaires were administered. These were subsequently screened, and responses with invalid patterns were excluded. Multivariate outliers were identified using Mahalanobis distance. Cases exceeding the critical *χ*^2^ value corresponding to the number of observed variables at *p* < 0.001 were considered multivariate outliers and excluded from subsequent analyses (Tabachnick & Fidell, 2019). Seven cases met this criterion and were removed prior to conducting the psychometric analyses.

Given that the two-factor structure of the General Belongingness Scale (GBS) has been consistently established in previous validation studies (Malone et al., 2012; Checa & Oberst, 2022; Yildiz, 2017), the present study adopted a confirmatory approach. Accordingly, confirmatory factor analysis (CFA) was conducted to evaluate the fit of the hypothesised measurement model in a sample of Spanish adolescents. Given that multivariate normality was not supported in the pre-analytical screening, the Maximum Likelihood estimation method with bootstrap correction (5000 resamples) was applied. Model fit was evaluated using multiple indices: the Root Mean Square Error of Approximation (RMSEA), with values < 0.08 indicating acceptable fit and <0.06 excellent fit; the Comparative Fit Index (CFI) and Tucker–Lewis Index (TLI), where values ≥ 0.90 denote acceptable fit and ≥0.95 good fit; and the Standardised Root Mean Square Residual (SRMR), with scores ≤ 0.08 considered acceptable and ≤0.05 excellent (Brown, 2006; B. M. Byrne, 2015; Hu & Bentler, 1999). Additionally, the Akaike Information Criterion (AIC) and Bayesian Information Criterion (BIC) were computed to compare competing models.

Although the original GBS literature proposes a two-factor structure (Acceptance/Inclusion and Rejection/Exclusion), previous validation studies in Spanish adults have suggested alternative dimensional solutions due to potential methodological artefacts. Therefore, several competing models were tested, including M0—a model without factors, M1—a unidimensional model, M2—the original two-factor model, and M3—a model with two correlated factors. This comparative approach is consistent with recommendations for cross-cultural validation studies, aiming to determine whether the theoretical structure of the GBS is retained in the adolescent Spanish population (Brown, 2006; B. M. Byrne, 2015; S. M. Byrne et al., 2010).

A classical item analysis was subsequently performed to evaluate item performance. Parameters included item means, standard deviations, skewness, kurtosis, item–total correlations, adjusted item–total correlations, item–factor correlations, corrected item–factor correlations, and the change in Cronbach’s alpha if the item were removed. Internal consistency was assessed for the total scale and subdimensions using Cronbach’s alpha and McDonald’s Omega, with coefficients ≥ 0.70 considered acceptable (Hayes & Coutts, 2020).

Next, the configural, metric, and scalar factorial invariance of the GBS across gender was examined using Multigroup Confirmatory Factor Analysis (MGCFA). A hierarchical approach was applied, beginning with a baseline (configural) model, followed by progressively restrictive models testing equality of factor loadings (metric invariance) and intercepts (scalar invariance). Invariance was evaluated through the adjusted Chi-square difference test (Δ*χ*^2^, *p* > 0.05), ΔCFI ≤ 0.01, and ΔRMSEA ≤ 0.015 (Asparaouhov & Muthén, 2014; Chen, 2007; Cheung & Rensvold, 2002). Once at least scalar invariance was established, latent mean differences were examined by fixing the latent mean of boys to zero and freely estimating that of girls; statistical significance was determined via *z*-values exceeding |1.96| (B. M. Byrne, 2015).

The relationship between students’ perceived belongingness (GBS total score) and school refusal behaviour (SCREEN dimensions) was first explored using Pearson’s bivariate correlation coefficients. Effect sizes were interpreted following Cohen’s (1988) criteria: small (0.10–0.29), moderate (0.30–0.49), and large (≥0.50).

To identify distinct subgroups of adolescents characterised by different levels of perceived belongingness, a Latent Profile Analysis (LPA) was performed. Competing models with an increasing number of latent profiles were estimated, and the optimal solution was selected based on theoretical interpretability and the following statistical criteria: lower AIC and BIC values, entropy values close to 1, and statistically significant Vuong-Lo-Mendell-Rubin Likelihood Ratio Tests (VLMR-LRT) (*p* < 0.05) (Song & Kim, 2019). Profiles containing fewer than 25 participants were discarded to ensure reliable classification. Interpretation of latent profiles was based on standardised scores: values below −0.50 were considered low, scores between −0.50 and 0.50 average, and values above 0.50 high (Gonzálvez et al., 2019). After identifying the optimal latent profile solution, gender and age differences in the distribution of GBS profiles were analysed using Chi-square tests.

Finally, differences in school refusal dimensions (SCREEN subscales) across the GBS latent profiles were examined using a Multivariate Analysis of Variance (MANOVA). When significant multivariate effects were detected, Bonferroni-adjusted post hoc tests were conducted to explore pairwise differences. Effect sizes were reported using Cohen’s *d*, interpreted as small (0.20–0.49), moderate (0.50–0.79), or large (≥0.80) (Cohen, 1988).

All analyses were conducted using IBM SPSS Statistics (version 28.0), AMOS software (version 28.0), and Mplus (version 8.10).

## 3. Results

### 3.1. Confirmatory Factor Analysis (CFA)

Confirmatory Factor Analysis (CFA) was conducted to examine the factorial structure of the General Belongingness Scale (GBS) in Spanish adolescents and to compare alternative competing models. Table 2 presents the fit indices for the four tested models. As expected, the null model (M0) exhibited a very poor fit to the data (*χ*^2^ = 2501.76, df = 66, *p* < 0.001), with unacceptable values across all indices (RMSEA = 0.231; SRMR = 0.308; CFI = 0.000; TLI = 0.000), confirming the presence of substantial shared variance among items.

The unidimensional model (M1) showed an improvement in fit compared to the null model; however, results remained clearly below recommended thresholds (*χ*^2^ = 513.15, df = 54, *p* < 0.001; RMSEA = 0.111 [0.102–0.120]; SRMR = 0.077; CFI = 0.811; TLI = 0.770). Likewise, the two-factor structure without inter-factor correlation (M2) offered a slightly better solution (*χ*^2^ = 368.44, df = 54, *p* < 0.001; RMSEA = 0.092 [0.083–0.101]; TLI = 0.842; CFI = 0.871), yet it still did not reach acceptable global fit levels, and the SRMR remained notably high (0.178), suggesting that constraining the two dimensions to be orthogonal does not adequately represent the underlying structure.

In contrast, the two-factor correlated model (M3), distinguishing Inclusion/Acceptance and Exclusion/Rejection, showed a substantial improvement and provided the best representation of the data. This model achieved good overall fit (*χ*^2^ = 156.49, df = 53, *p* < 0.001; *χ*^2^/df = 2.95; RMSEA = 0.053 [0.044–0.063]; SRMR = 0.039; CFI = 0.958; TLI = 0.947) alongside the lowest AIC (206.491) and BIC (319.908) values among all tested models. These indices collectively indicate that allowing the two factors to correlate substantially enhances model performance and accurately captures the latent structure of the GBS in adolescents.

### 3.2. Descriptive Analysis of the Items and the Scales

Table 3 presents the descriptive statistics and item-level indices for the 12 items of the General Belongingness Scale (GBS), including means, standard deviations, skewness and kurtosis, as well as item–test correlations and the effect on Cronbach’s alpha when each item was removed. Item means ranged from 1.01 (item 12) to 3.65 (item 8), whereas standard deviations varied between 1.30 (item 3) and 1.50 (item 5). The values of Cronbach’s alpha if an item were deleted ranged from 0.73 (items 3 and 7) to 0.81 (item 2), indicating that the removal of any item would not meaningfully improve the internal consistency of the scale.

Skewness coefficients ranged from –1.05 (item 8) to 1.35 (item 12), reflecting acceptable deviations from normality for self-report data. Kurtosis values ranged from –0.60 (item 9) to 1.02 (item 12), also within recommended limits for psychometric analyses. Item–test correlations showed adequate discriminative capacity, with coefficients ranging from 0.60 (item 12) to 0.77 (items 10 and 11), all exceeding the 0.50 standard commonly used to indicate satisfactory item quality.

At the subscale level, the Inclusion dimension demonstrated good internal consistency (α = 0.81, Ω = 0.81; composite reliability = 0.82), with a mean of 20.18 (*SD* = 5.91), indicating that it is the most prevalent factor in the Spanish sample. Similarly, the Exclusion dimension showed adequate reliability (α = 0.78, Ω = 0.78; composite reliability = 0.79), with a mean of 7.85 (*SD* = 5.61).

### 3.3. Factorial Invariance Across Gender

A series of multigroup confirmatory factor analyses (MGCFA) was conducted to examine whether the two-factor structure of the GBS operates equivalently for boys and girls (see Table 4). The initial configural model (M0), in which the same factorial structure was specified for both groups with freely estimated parameters, demonstrated an adequate fit to the data (*χ*^2^/*df* = 2.183; TLI = 0.936; CFI = 0.948; RMSEA = 0.042 [0.034–0.049]), indicating that boys and girls conceptualise the underlying constructs in a comparable manner.

Building upon this baseline model, metric invariance (M1) was tested by constraining factor loadings to equality across gender. Fit indices remained satisfactory (*χ*^2^/*df* = 2.011; TLI = 0.945; CFI = 0.952; RMSEA = 0.039 [0.031–0.046]), and comparison with the configural model yielded negligible differences (ΔCFI = 0.003; ΔRMSEA = 0.003; Δ*χ*^2^ = 1.876, *p* = 0.997). These results support the equivalence of factor loadings for boys and girls, indicating that the items relate to the latent factors with similar strength across gender.

The scalar invariance model (M2), which additionally constrained item intercepts, also demonstrated an acceptable fit (*χ*^2^/*df* = 2.125; TLI = 0.939; CFI = 0.943; RMSEA = 0.041 [0.034–0.047]). Model comparisons revealed no meaningful deterioration in fit relative to the metric model (ΔCFI = 0.009; ΔRMSEA = 0.002), and the chi-square difference test approached but did not reach statistical significance (Δ*χ*^2^ = 15.007, *p* = 0.056). This pattern of results indicates that scalar invariance can be reasonably assumed, allowing for meaningful comparison of latent means between boys and girls.

Finally, strict invariance (M3), which further constrained residual variances, was assessed. Although overall fit indices remained acceptable (TLI = 0.938; CFI = 0.936; RMSEA = 0.041 [0.035–0.047]), comparison with the scalar model indicated a statistically significant *χ*^2^ difference (Δ*χ*^2^ = 44.249, *p* < 0.001) and a small but notable decrease in CFI (ΔCFI = 0.007). Given that strict invariance is often considered overly restrictive and rarely achieved in practice, and in line with recommendations to prioritise configural, metric, and scalar levels when establishing invariance (Yuan & Bentler, 2004; Dimitrov, 2010), these results support the interpretation that the GBS demonstrates partial strict invariance across gender.

Taken together, the findings indicate that the GBS functions equivalently for boys and girls at the configural, metric, and scalar levels, enabling valid comparisons of latent factor means across gender groups.

### 3.4. Correlation Coefficients Between the GBS and the Screen

Pearson’s correlations were calculated to examine the associations between the GBS dimensions and the four SCREEN factors (see Table 5). The Inclusion dimension showed small-to-moderate negative correlations with all SCREEN factors (from *r* = −0.08 to −0.32). By contrast, Exclusion displayed small-to-moderate positive associations with the SCREEN factors (from *r* = 0.16 to *r* = 0.40). All coefficients, except for the association between Inclusion and Difficult Transition, were statistically significant.

### 3.5. Belongingness Profiles

A Latent Profile Analysis (LPA) was conducted to identify distinct socio-emotional patterns of belongingness based on the Inclusion and Exclusion dimensions of the GBS. Model fit indices for the two- to six-profile solutions are presented in Table 6. Although the five- and six-profile models yielded lower AIC and BIC values, these solutions were discarded due to suboptimal entropy values (0.748 and 0.735, respectively) and the presence of clusters with fewer than 25 participants, indicating insufficient subgroup stability. The two-profile model was also rejected, as it showed the highest AIC and BIC values and comparatively weak entropy (0.701).

Among the remaining models, the four-profile solution demonstrated the most favourable balance of statistical adequacy and substantive interpretability. This model showed comparatively low AIC and BIC values (AIC = 3153.264; BIC = 3211.836), the highest entropy (0.825), and statistically significant likelihood-ratio tests (LRT, adjusted LRT and BLRT all *p* < 0.001), indicating clear distinction between latent classes. Additionally, all four clusters met recommended sample size criteria and showed coherent psychological profiles, further supporting the selection of the four-profile model as the optimal representation of the data.

**Figure 1:**
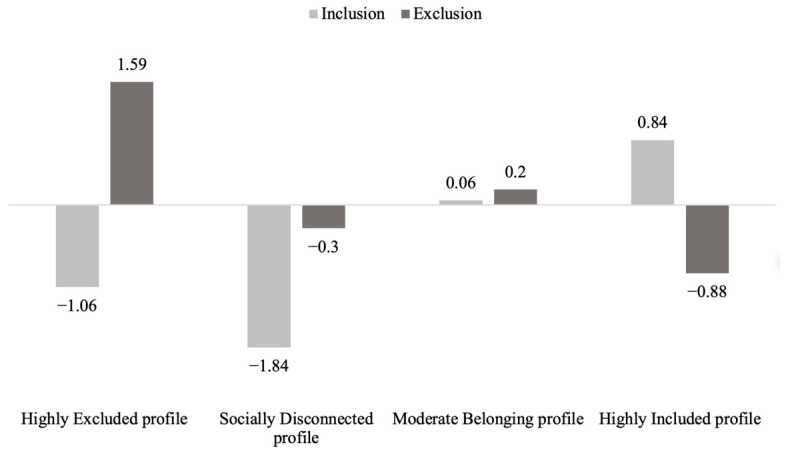
Belonging profiles.

Figure 1 shows the *z*-standardised means for Inclusion and Exclusion across the four profiles. The first profile, labelled Highly Excluded (*n* = 119; 17.2%), was characterised by markedly low Inclusion (*z* = −1.05) and very high Exclusion (*z* = 1.59). The second profile, Socially Disconnected (*n* = 56; 8.1%), exhibited very low Inclusion (*z* = −1.84) combined with slightly below-average Exclusion (*z* = –0.30), indicating adolescents who feel neither actively included nor overtly rejected. The third profile, Mixed Belonging (*n* = 260; 37.7%), reflected scores close to the sample mean on both dimensions (Inclusion *z* = 0.06; Exclusion *z* = 0.20), representing adolescents who experience a mixed pattern of belongingness, characterised by relatively average levels of both inclusion and exclusion, without a clearly positive or negative interpersonal orientation. Finally, the fourth profile, Highly Included (*n* = 255; 37%), showed high Inclusion (*z* = 0.83) and low Exclusion (*z* = −0.88), capturing adolescents who perceive strong integration and minimal rejection within their social contexts.

Across the distribution, four belongingness profiles (Table 7), gender distribution was generally balanced, with no statistically significant differences between boys and girls (χ^2^ = 5.26, *p* = 0.154). The Highly Excluded group (n = 117) included 50 boys and 67 girls, while the Socially Disconnected profile (n = 55) comprised 33 boys and 22 girls. The two largest profiles, Mixed Belonging (*n* = 256) and Highly Included (*n* = 253), displayed near-equal representation of boys and girls (132 vs. 124 and 133 vs. 120, respectively). A similar pattern was observed across age groups, with all profiles including adolescents aged 12 to 17 years and no significant age-related differences emerging (*χ*^2^ = 16.01, *p* = 0.382). These findings indicate that the belongingness profiles are demographically comparable across gender and age.

### 3.6. Belongingness Profiles and School Refusal

Table 8 and Table 9 summarise the differences in SCREEN factor scores across the four belongingness profiles. The MANOVA indicated significant multivariate differences among profiles on the four SCREEN dimensions (Wilks’ λ = 0.83, *F*_(3, 686)_ = 10.86, *p* < 0.001, η_p_^2^ = 0.06). Overall, adolescents in the Highly Excluded profile demonstrated the highest levels of school refusal across all dimensions, whereas those in the Highly Included profile consistently exhibited the lowest mean scores.

For Anxious Anticipation (FI), the Highly Excluded group showed the highest average score (*M* = 3.58), followed by the Socially Disconnected (*M* = 2.51), Mixed Belonging (*M* = 1.76), and Highly Included (*M* = 1.03) profiles. Post hoc comparisons revealed several significant contrasts: medium to large effect sizes emerged between Highly Excluded and the remaining profiles (*d* = 0.37 to *d* = 1.15), with the largest difference observed relative to the Highly Included profile (*d* = 1.15). Significant differences also emerged between Socially Disconnected and Highly Included adolescents (*d* = 0.72), and between Mixed Belonging and Highly Included profiles (*d* = 0.36).

For Difficult Transition (FII), mean scores followed a similar pattern, with the Highly Excluded group again reporting the highest score (*M* = 6.89) and the Highly Included group the lowest (*M* = 5.84). Although few contrasts reached statistical significance, small effect sizes were noted between Highly Excluded and Socially Disconnected adolescents (*d* = 0.43), and between Highly Excluded and Highly Included peers (*d* = 0.29).

Regarding Interpersonal Discomfort (FIII), the Highly Excluded profile obtained the highest mean score (*M* = 5.82), whereas the Highly Included group showed the lowest (*M* = 3.81). Post hoc tests indicated moderate effect sizes between Highly Excluded and Socially Disconnected (*d* = 0.61), Mixed Belonging (*d* = 0.41), and Highly Included adolescents (*d* = 0.68). A small but significant contrast also emerged between Mixed Belonging and Highly Included profiles (*d* = 0.25).

Finally, for School Avoidance (FIV), the Highly Excluded profile again presented the highest mean score (*M* = 3.14), followed by Socially Disconnected (*M* = 2.75), Mixed Belonging (*M* = 2.09), and Highly Included (*M* = 1.55) adolescents. Significant differences with small-to-moderate effect sizes were observed between Highly Excluded and Mixed Belonging (*d* = 0.40), Highly Excluded and Highly Included (*d* = 0.69), and between Socially Disconnected and Highly Included adolescents (*d* = 0.56).

## 4. Discussion

The present study had two major aims: (1) to validate the General Belongingness Scale (GBS; Malone et al., 2012) in Spanish adolescents and (2) to identify latent belongingness profiles and examine how these socio-emotional configurations relate to school refusal. Overall, the findings provide strong support for the psychometric adequacy of the GBS in this population and reveal meaningful heterogeneity in adolescents’ belongingness experiences that is systematically associated with school refusal behaviours.

Regarding the first objective, the CFA results confirmed that the GBS retains a two-factor structure, Acceptance/Inclusion and Rejection/Exclusion, in Spanish adolescents. This finding is consistent with the original validation (Malone et al., 2012) and with prior evidence in young adults and adolescents from other cultural contexts (Satici & Gocet-Tekin, 2016; Yildiz, 2017), supporting Hypothesis 1 and reinforcing the conceptualisation of belongingness as comprising two closely related yet distinguishable dimensions.

Notably, this result diverges from the Spanish adult validation by Checa and Oberst (2022), who reported a one-factor solution. Several developmental and methodological explanations may account for this discrepancy. First, adolescence is a developmental period characterised by heightened sensitivity to social inclusion and social threat, with stronger differentiation between experiences of acceptance and rejection (Allen & Bowles, 2012; Gallé-Tessonneau et al., 2019). Thus, adolescents may perceive inclusion and exclusion as more distinct emotional and relational states than adults, who tend to integrate social experiences into a more global sense of connectedness (Checa & Oberst, 2022). Second, adults might show reduced variability in exclusion experiences compared with adolescents, leading to stronger factor overlap and the emergence of a dominant general factor. Overall, the current findings provide empirical support for the multidimensional nature of belongingness during adolescence, coherently integrating theoretical predictions and developmental considerations.

The classical item analysis reinforced the structure’s robustness, as all items demonstrated adequate distributional properties, discriminative power and contributions to internal consistency. Both dimensions showed good reliability (α = 0.81 for Inclusion; α = 0.78 for Exclusion), comparable to values reported in Turkish and American studies (Malone et al., 2012; Yildiz, 2017), supporting Hypothesis 2. Importantly, the reliability indices in adolescents were similar to those obtained in adults in Spain (Checa & Oberst, 2022), a pattern expected given developmental differences in self-concept consolidation (Gallé-Tessonneau et al., 2019).

Measurement invariance tests further confirmed that the scale functions equivalently for boys and girls at the configural, metric and scalar levels, allowing direct comparison of latent means across gender. This finding aligns with the Spanish adult validation (Checa & Oberst, 2022) and supports the gender generalizability of the GBS in Spain, supporting Hypothesis 3. Partial strict invariance, although not fully satisfied, is common in psychological measures, especially in adolescence, and does not undermine the validity of latent mean comparisons (Dimitrov, 2010).

Convergent validity results were also theoretically coherent. Inclusion correlated negatively with the four SCREEN dimensions, whereas Exclusion correlated positively, reflecting the strong links between belongingness and internalising manifestations such as anxiety, social discomfort and avoidance tendencies (Hamilton, 2024; Yildirim et al., 2023). Although correlations were small to moderate, this magnitude is consistent with the conceptual distinction between general belongingness and school-specific emotions (Heyne et al., 2019; Havik & Ingul, 2022), supporting Hypothesis 4.

Beyond measurement validity, the study identified four distinct belongingness profiles: Highly Excluded, Socially Disconnected, Mixed Belonging and Highly Included. This typology highlights considerable interpersonal variability that cannot be captured through variable-centred analyses. Importantly, the profiles aligned with theoretically meaningful gradients of psychosocial functioning (Gonzálvez et al., 2019). The Highly Excluded group showed markedly low inclusion and very high exclusion, consistent with patterns of social withdrawal, negative affect and heightened relational sensitivity that characterise adolescents at risk for internalising problems (Allen & Bowles, 2012; Gonzálvez et al., 2019; Yildiz, 2016). Conversely, the Highly Included group displayed strong inclusion and low exclusion, reflecting a protective socio-emotional configuration associated with social integration, attachment security and well-being (Yildiz, 2017; Yildirim et al., 2023), supporting Hypothesis 5.

A central contribution of this study lies in its examination of how belongingness profiles relate to school refusal, a domain where empirical evidence has been notably absent despite strong theoretical justification (Hamilton, 2024). The MANOVA and post hoc tests revealed a clear and coherent gradient: adolescents in the Highly Excluded profile reported the highest levels of anxious anticipation, interpersonal discomfort, difficult transitions, and school avoidance. In contrast, the Highly Included group consistently showed the lowest levels across all four SCREEN factors. The Socially Disconnected profile with low inclusion but not high rejection represented an intermediate, more ambiguous risk pattern, consistent with adolescents who feel invisible rather than actively rejected. These findings align with theoretical models proposing that low belongingness increases risk for school refusal through emotional distress, peer difficulties and avoidance-based coping (Gonzálvez et al., 2019; Gubbels et al., 2019; Hamilton, 2024; Leduc et al., 2024). They also strengthen the argument that belongingness may play a preventive role, buffering adolescents against internalising symptoms that precede school refusal (Gonzálvez et al., 2018; Yildirim et al., 2023).

An additional mechanism that may contribute to the association between belongingness and school refusal is peer victimisation. Adolescents who experience bullying are more likely to report feelings of rejection, social exclusion, and reduced school belonging, all of which have been associated with increased emotional distress and school avoidance (Arslan & Allen, 2021; Kljakovic et al., 2021). Although bullying was not assessed in the present study, it may represent an important contextual factor linking low belongingness to school refusal. Future research should therefore examine whether experiences of peer victimisation mediate or moderate the relationship between belongingness profiles and school refusal behaviours.

Beyond individual perceptions of belongingness, the present findings also underscore the broader significance of school climate in shaping adolescents’ socio-emotional functioning and school attendance patterns (Pérez-Marco et al., 2025a). A supportive school climate, characterised by positive peer relationships, teacher responsiveness, fairness, safety, and opportunities for participation, has consistently been shown to strengthen students’ sense of belonging and to reduce emotional and behavioural difficulties (Cohen et al., 2009; Cunningham et al., 2022; Fernández-Sogorb et al., 2026). The marked differences observed between the Highly Included and Highly Excluded profiles in school refusal behaviour reflect these dynamics. While adolescents who experience inclusion likely benefit from warmer interpersonal climates, greater feelings of safety and trust, and more supportive interactions, all of which buffer anxiety and reduce avoidance tendencies. Conversely, students who report heightened exclusion may perceive their school environment as less predictable, less caring, or even threatening, conditions known to exacerbate interpersonal discomfort, negative affect, and withdrawal behaviours. These results align with ecological models (Kearney, 2008), emphasising that belongingness emerges not only from individual relationships but also from the quality of the broader school context. Strengthening school climate may therefore serve as a structural level for enhancing belongingness and, in turn, preventing the development or escalation of school refusal (Cunningham et al., 2022; Fernández-Sogorb et al., 2026; Pérez-Marco et al., 2025a).

To the authors’ knowledge, no previous studies have examined that configurations of belongingness meaningfully differentiate adolescents in their vulnerability to school refusal, thereby providing the novel empirical support to Hamilton’s (2024) theoretical model. These insights highlight the value of integrating belongingness into school-based early-warning systems and intervention programmes.

## 5. Conclusions

This study provides the validation of the GBS in Spanish adolescents and introduces a novel, person-centred understanding of belongingness in this population. Nevertheless, some limitations warrant consideration. First, the use of self-report measures may introduce biases, although this is common and unavoidable in socio-emotional constructs (Baumeister et al., 2007). Second, the sample consisted of adolescents from a single cultural context, limiting cross-national generalisability. Future research should replicate the latent profile structure in other Spanish-speaking regions. Furthermore, the number of participants in certain age-by-gender subgroups (particularly 17-year-old boys and girls) was smaller than the rest of the groups, limiting the robustness of age-specific analyses. Third, although the study demonstrated meaningful associations between belongingness and school refusal, causal inferences cannot be drawn; longitudinal designs are highly recommended. In addition, although a small proportion of participants identified as another gender, this group was not included in measurement invariance analyses due to insufficient sample size for reliable multi-group comparisons (Putnick & Bornstein, 2016). Future research should aim to include larger and more diverse gender-diverse samples to allow for meaningful invariance testing across gender identities beyond the binary classification. Finally, although LPA identified distinct profiles, mixed-methods approaches could enrich understanding of how adolescents interpret belongingness within their relational environments.

Despite these limitations, the findings have clear practical implications. First, the validated GBS offers researchers and practitioners a culturally appropriate and psychometrically robust tool for assessing belongingness in Spanish adolescents, a construct central to socio-emotional development. Since a higher sense of belongingness is associated with lower school refusal, educators and school psychologists should consider assessing belongingness as part of their evaluation of at-risk students in schools. The Spanish GBS can be used as a diagnostic tool to identify students with low belongingness in schools. Second, the identification of four belongingness profiles provides a framework for targeted, profile-based interventions. Interventions such as peer-support programmes, mentoring, and inclusive classroom practices could then be targeted to those students to enhance their school belonging. Specifically, students in the Highly Excluded and Socially Disconnected profiles may particularly benefit from programmes enhancing peer connectedness, social-emotional skills and supportive school climate (Pérez-Marco et al., 2025a). Third, given the strong associations between belongingness and school refusal, promoting belongingness may serve as an effective early-intervention strategy to reduce emotionally driven school avoidance, complementing existing approaches focused on anxiety or family dynamics (Heyne et al., 2019; Pérez-Marco et al., 2025b).

In summary, this study expands empirical knowledge on adolescent belongingness, introduces the first validated GBS for Spanish youth, and demonstrates how belongingness configurations shape vulnerability to school refusal. By integrating psychometric validation with person-centred modelling, the findings offer a comprehensive framework that can inform both research and applied practice in educational and clinical settings.

## Figures and Tables

**Table 1:** Sample distribution by gender and age.

Gender	12 Years	13 Years	14 Years	15 Years	16 Years	17 Years	Total
Boys	44 6.4%	90 13%	84 12.2%	60 8.7%	51 7.4%	19 2.8%	348 50.4%
Girls	38 5.5%	70 10.1%	71 10.3%	79 11.4%	54 7.8%	21 3%	333 48.3%
Another	0 0%	1 0.1%	6 0.9%	1 0.1%	1 0.1%	0 0%	9 1.3%
Total	82 11.9%	161 23.3%	161 23.3%	140 20.3%	106 15.4%	40 5.8%	690 100%

**Table 2:** Confirmatory factor analyses: goodness-of-fit indices of the statistic models of the GBS.

Model	*χ* ^2^	df	*p*	*χ*^2^/df	RMSEA 90% I.C	SRMR	TLI	CFI	AIC	BIC
M0	2501.760	66	<0.001	37.905	0.231 (0.224–0.239)	0.308	0.000	0.000	2525.760	2580.201
M1	513.147	54	<0.001	9.503	0.111 (0.102–0.120)	0.077	0.770	0.811	561.147	670.027
M2	368.441	54	<0.001	6.823	0.092 (0.083–0.101)	0.178	0.842	0.871	416.441	525.321
**M3**	**156.491**	**53**	**<0.001**	**2.953**	**0.053 (0.044–0.063)**	**0.039**	**0.947**	**0.958**	**206.491**	**319.908**

Note: M0 = Without factors; M1 = Model with one factor; M2 = Authors’ model; M3 = Model of four correlated factors; *χ*^2^ = Chi squared; *df* = Degrees of freedom; RMSEA = Root means square error of approximation; SRMR = Standardised root mean square residual; TLI = Tucker–Lewis coefficient; CFI = Comparative fit index; AIC = Akaike Information Criterion; BIC = Bayesian Information Criterion; row in bold = selected model.

**Table 3:** Statistics for each dimension and item of the GBS model.

Items	*M*	*SD*	*A*	*K*	*FL*	*R_IS_*	*R_ISc_*	α-it
FI: Inclusion *M* = 20.18; *SD* = 5.91; α = 0.81; Ω = 0.81; Composted reliability: 0.82								
GBS1	3.14	1.34	−0.55	−0.37	0.58	0.68	0.53	0.79
GBS2	3.61	1.50	−1.02	0.07	0.48	0.64	0.45	0.81
GBS5	3.23	1.31	−0.69	−0.10	0.66	0.70	0.56	0.78
GBS8	3.65	1.39	−1.05	.35	0.70	0.76	0.62	0.77
GBS10	3.33	1.36	−0.72	−0.15	0.72	0.77	0.64	0.77
GBS11	3.22	1.34	−0.76	−0.07	0.75	0.77	0.65	0.77
FII: Exclusion *M* = 7.85; *SD* = 5.612; α = 0.78; Ω = 0.78; Composted reliability: 0.79								
GBS3	1.45	1.30	0.78	−0.01	0.68	0.74	0.60	0.73
GBS4	1.55	1.45	0.77	−0.31	0.64	0.71	0.54	0.74
GBS6	1.08	1.32	1.21	0.68	0.66	0.72	0.57	0.74
GBS7	1.11	1.31	1.20	0.79	0.73	0.75	0.62	0.73
GBS9	1.64	1.42	0.58	−0.60	0.50	0.63	0.43	0.77
GBS12	1.01	1.32	1.35	1.02	0.48	0.60	0.41	0.77

Note: *M* = Mean; *SD* = Standard Deviation; *A* = Asymmetry; *K* = Kurtosis; *FL* = Standardised Factor Loadings; *R_IS_* = correlation item-scale; *R_ISc_* = correlation item-corrected scale; α-it = Cronbach’s alpha if the item were deleted.

**Table 4:** Goodness-of-fit indices for the own model of the GBS depending on gender.

Gender	*χ* ^2^	*df*	*χ*^2^/*df*	TLI	CFI	RMSEA	Δ*χ*^2^(*p*)	ΔRMSEA	ΔCFI
Boys	89.472	53	1.688	0.960	0.968	0.045 (0.028–0.060)			
Girls	141.958	53	2.678	0.915	0.932	0.071 (0.057–0.085)			
M0	231.434	106	2.183	0.936	0.948	0.042 (0.034–0.049)			
M1	233.311	116	2.011	0.945	0.952	0.039 (0.031–0.046)	1.876 (0.997)	0.003	0.003
M2	248.318	128	2.125	0.939	0.943	0.041 (0.034–0.047)	15.007 (0.056)	0.002	0.009
M3	294.267	143	2.135	0.938	0.936	0.041 (0.035–0.047)	44.249 (<0.001)	0.000	0.007

Note: M0 = free model (configural invariance); M1 = Model 0 with factorial loads (metric invariance); M2 = Model 1 with intercepts (scalar invariance); M3 = Model 2 with error variance (strict invariance); *χ*^2^ = Chi squared; *df* = Degrees of freedom; TLI = Tucker–Lewis Index; RMSEA = Root mean square error of approximation; CFI = Comparative fit index.

**Table 5:** Correlations among Belongingness and School Refusal.

Dimensions	Inclusion	Exclusion
Anxious anticipation	–0.32 **	0.40 **
Difficult transition	–0.01	0.16 **
Interpersonal discomfort	–0.08 *	0.30 **
School avoidance	–0.21 **	0.28 **

Note: * *p* < 0.05; ** *p* < 0.001.

**Table 6:** Indices of fit for Latent Profile Analysis.

Models	AIC	BIC	BIC-Adjusted	LRT *p*	LRT-Adjusted	BLRT	Entropy	Size
2	3256.972	3292.945	3271.709	<0.001	<0.001	<0.001	0.701	0
3	3184.222	3231.474	3202.715	<0.001	<0.001	<0.001	0.816	0
**4**	**3153.264**	**3211.836**	**3174.616**	**<0.001**	**<0.001**	**<0.001**	**0.825**	**0**
5	3126.651	3190.474	3159.685	0.128	0.154	<0.001	0.748	1
6	3109.845	3198.957	3139.633	0.224	0.239	<0.001	0.735	1

Note: AIC = Akaike Information Criteria; BIC = Bayesian Information Criteria; LRT = Vuong-Lo- Mendell-Rubin Likelihood-Ratio Test; BLRT = Bootstrap Likelihood Ratio Test; Bold data = selected model.

**Table 7:** Distribution of belonging profiles across gender and age.

Profiles	Total	Gender		Age					
		Boy	Girl	12	13	14	15	16	17
		*χ*^2^ = 5.26; *p* = 0.154		*χ*^2^ = 16.01; *p* = 0.382					
Highly excluded	117	50	67	11	26	20	21	28	11
	17.2%	14.4%	20.1%	13.4%	16.3%	12.9%	15.1%	26.7%	27.5%
Socially Disconnected	55	33	22	6	15	13	12	6	3
	8.1%	9.5%	6.6%	7.3%	9.4%	8.4%	8.6%	5.7%	7.5%
Mixed Belonging	256	132	124	29	62	62	50	38	15
	37.6%	37.9%	37.2%	35.4%	38.8%	40%	36%	36.2%	37.5%
Highly included	253	133	120	36	57	60	56	33	11
	37.2%	38.2%	36%	43.9%	35.6%	38.7%	40.3%	31.4%	27.5%
Total	681	348	333	82	160	155	139	105	40
	100%	100%	100%	100%	100%	100%	100%	100%	100%

**Table 8:** Means and standard deviations obtained by the four groups in the dimensions of SCREEN.

Dimensions	Highly Excluded (N = 119)		Socially Disconnected (N = 56)		Mixed Belonging (N = 260)		Highly Included (N = 255)		Statistical Significance		
	M	DT	M	DT	M	DT	M	DT	*F*_(2, 1127)_	*p*	*η* ^2^
Anxious anticipation	3.58	2.99	2.51	2.73	1.76	2.24	1.03	1.76	35.46	<0.001	0.13
Difficult transition	6.89	3.57	5.37	3.38	6.05	3.35	5.84	3.62	3.28	<0.001	0.01
Interpersonal discomfort	5.82	3.29	3.94	2.54	4.55	3.05	3.81	2.76	13.17	<0.001	0.05
School avoidance	3.14	2.87	2.75	2.71	2.09	2.46	1.55	2.00	13.18	<0.001	0.05

**Table 9:** Cohen’s d value for post hoc values between cluster groups in SCREEN.

Dimensions		HE-SD	HE-MB	HE-HI	SD-MB	SD-HI	MB-HI
Anxious anticipation	*p*	0.025	<0.001	<0.001	*n. s.*	<0.001	0.002
	*d*	0.37	0.73	1.15	*-*	0.72	0.36
Difficult transition	*p*	0.046	*n. s.*	0.043	*n. s.*	*n. s.*	*n. s.*
	*d*	0.43	*-*	0.29	*-*	*-*	*-*
Interpersonal discomfort	*p*	0.001	0.001	<0.001	*n. s.*	*n. s.*	0.026
	*d*	0.61	0.41	0.68	*-*	*-*	0.25
School avoidance	*p*	*n. s.*	0.001	<0.001	*n. s.*	0.005	*n. s.*
	*d*	*-*	0.40	0.69	*-*	0.56	*-*

Note: HE: Highly Excluded profile; SD: Socially Disconnected profile; MB: Mixed Belonging profile; HI: Highly Included profile; *n. s.*: not significant.

## Data Availability

The data will be available on request.
